# Association between grip strength and non-alcoholic fatty liver disease: A systematic review and meta-analysis

**DOI:** 10.3389/fmed.2022.988566

**Published:** 2022-08-26

**Authors:** Liu Han, Shifeng Fu, Jianglei Li, Deliang Liu, Yuyong Tan

**Affiliations:** ^1^Department of Gastroenterology, The Second Xiangya Hospital of Central South University, Changsha, China; ^2^Research Center of Digestive Disease, Central South University, Changsha, China

**Keywords:** NAFLD, grip strength, review, meta-analysis, observational study

## Abstract

**Background:**

The association between grip strength (GS) and non-alcoholic fatty liver disease (NAFLD) has been reported by recent epidemiological studies, however, the results of these studies are inconsistent. This meta-analysis was conducted to collect all available data and estimate the risk of NAFLD among people with low GS, as well as the risk of low GS among patients with NAFLD.

**Methods:**

We systematically searched several literature databases including PubMed, Web of Science, Cochrane Library, and Embase from inception to March 2022. These observational studies reported the risk of NAFLD among people with low GS and/or the risk of low GS among patients with NAFLD. Qualitative and quantitative information was extracted, statistical heterogeneity was assessed using the *I*^2^ test, and potential for publication bias was assessed qualitatively by a visual estimate of a funnel plot and quantitatively by calculation of the Begg’s test and the Egger’s test.

**Results:**

Of the citations, 10 eligible studies involving 76,676 participants met inclusion criteria. The meta-analysis of seven cross-section studies (69,757 participants) showed that people with low GS had increased risk of NAFLD than those with normal GS (summary OR = 3.32, 95% CI: 1.91–5.75). In addition, the meta-analysis of four studies (14,920 participants) reported that the risk of low GS patients with NAFLD was higher than those in normal people (summary OR = 3.31, 95% CI: 2.45–4.47).

**Conclusion:**

In this meta-analysis, we demonstrated a strong relationship between low GS and NAFLD. We found an increased risk of NAFLD among people with low GS, and an increased risk of lower GS among NAFLD patients.

**Systematic review registration:**

[www.crd.york.ac.uk/prospero], identifier [CRD42022334687].

## Introduction

Currently, non-alcoholic fatty liver disease (NAFLD) has become one of the most common causes of chronic liver disease, it is defined by the presence of steatosis in more than 5% of hepatocytes with little or no alcohol consumption (1). NAFLD is characterized by fatty infiltration of the liver without secondary causes of hepatic steatosis (2).

In the United States, approximately 30% of individuals are diagnosed with NAFLD (3). In addition, over 27% of individuals are affected by NAFLD (4). In China, the prevalence of NAFLD was reported to be between 15 and 36% (5, 6). Additionally, as a result of the aging population and obesity, the prevalence of NAFLD is increasing rapidly. However, to date, there is no effective drug for treatment of NAFLD. As shown by a number of compelling studies, NAFLD is associated with some chronic diseases, such as type 2 mellitus (T2DM), cardiovascular disease (CVD), and chronic kidney disease (CKD) (7, 8). Therefore, understanding the pathobiology and risk factors for development of NAFLD is of great importance.

Grip strength (GS) is a measure of the maximum static force that a hand can apply around a dynamometer. GS is often considered an indicator of muscle mass and muscle strength (9). Researches have suggested that low GS is associated with health damage and higher all-cause mortality (10, 11), such as falls, disability and poor quality of life (12, 13). Indeed, previous studies have also shown association between NAFLD and sarcopenia (14). Low muscle strength is used as a principal determinant of sarcopenia over muscle mass (15), and GS is recommended as a substitute measurement of muscle strength (16). Therefore, in clinical practice, people are increasingly aware of the importance of muscle strength.

Non-alcoholic fatty liver disease is a systemic condition that has a bi-directional relationship with the components of metabolic syndrome (17). According to recent studies, muscular strength is inversely related to insulin sensitivity (18) and excessive body and abdominal fat (19), which are independent risk factors for developing NAFLD. Now, several studies have reported that association between GS and NAFLD, therefore, we collected these studies for meta-analysis as a way to explore the relationship between GS and NAFLD.

## Materials and methods

### Protocol and guidance

This meta-analysis followed the Preferred Reports Items for Systematic Reviews and Meta-analyses (PRISMA) reporting guideline (20). The protocol for this meta-analysis was registered with PROSPERO (CRD42022334687).

### Data sources and searches

Two investigators (LH and SF) independently conducted an electronic literature search using PubMed, Web of Science, Cochrane Library, and Embase, language was restricted to English, from database inception to March 2022. In PubMed, controlled vocabulary terms and the following keywords were used: (“Non-alcoholic Fatty Liver Disease” [Mesh]) OR (Non-alcoholic Fatty Liver Disease) OR (Non-alcoholic Fatty Liver Disease) OR (Fatty Liver, Non-alcoholic) OR (Fatty Livers, Non-alcoholic) OR (Liver, Non-alcoholic Fatty) OR (Livers, Non-alcoholic Fatty) OR (Non-alcoholic Fatty Liver) OR (Non-alcoholic Fatty Livers) OR (Non-alcoholic Steatohepatitis) OR (Non-alcoholic Steatohepatitides) OR (Steatohepatitides, Non-alcoholic) OR (Steatohepatitis, Non-alcoholic) AND (“Hand Strength” [Mesh]) OR (Strength, Hand) OR (Grip Strength) OR (Strength, Grip) OR (Hand Grip Strength) OR (Grip Strength, Hand) OR (Strength, Hand Grip) OR (Grip) OR (Grips) OR (Grasp) OR (Grasps). A similar search strategy was run in other databases. Supplementary Table 1 presents the search strategy.

The database search revealed 224 articles that could have been included in our meta-analysis, and 43 articles were excluded because they were duplicated. After removing duplicates, all titles and abstracts for potential inclusion were screened by two independent researchers (LH and SF). Based on the inclusion and exclusion criteria, 164 articles were excluded after reading the titles and abstracts. Finally, 17 full texts of these records were selected for detailed assessment. The two researchers extracted the related data according to the inclusion criteria. If the studies were potentially eligible for inclusion, the full text was examined. The two reviewers would discuss with each other any disagreements that may have occurred.

### Study quality assessment

All studies were assessed for selection and measurement biases according to the Newcastle-Ottawa Scale (NOS) (21). The NOS consists of eight items focused on three domains: selection of study groups, ascertainment of the exposure and outcome, and comparability of groups to assess the quality of observational studies. Ratings were based on a star system and studies with a maximum rating of nine. Studies with one to three stars were categorized as low quality, four to six stars categorized as moderate quality, and seven to nine stars categorized as high quality. Each of included studies was assessed for bias by two independent investigators (LH and SF).

### Inclusion criteria

The same two authors evaluated the titles and abstracts of eligible studies and any disagreements were resolved by consensus. The inclusion criteria are as follows: (1) studies on the association between NAFLD and GS; (2) used a standardized index to diagnose and assess NAFLD and GS; (3) reporting odds ratio (OR) and 95% confidence intervals (95% CI) for GS and NAFLD; (4) the full text of the study could be assessed; and (5) the full text of the study could be assessed.

### Exclusion criteria

The same two authors evaluated the titles and abstracts of eligible studies and any disagreements were resolved by consensus. The exclusion criteria are as follows: (1) did not use clear diagnostic criteria for NAFLD; (2) the measurement of GS is not accurate; (3) the study did not provide the OR of NAFLD and GS; (4) case reports, case series, reviews, posters, and abstracts were excluded; (5) measured only *in vitro* parameters or used animal models; and (6) based on the NOS scores, the low-quality studies were excluded.

### Data collection process

Data collection process two independent researchers (LH and SF) assessed the full texts of included studies and used a standard data extraction form when extracting data. Any disagreements were resolved by discussion until consensus was reached. The data extracted for the analysis involved: (1) the first author’s name and publication year; (2) the sample size and number of cases; (3) the mean age and sources of participants; (4) the OR with the corresponding 95% CI; and (5) the scores of NOS in the studies.

### Statistical analysis

The meta-analysis of comparable data was carried out using Review Manager 5.3. OR and their associated 95% CI were used to assess a comparison between outcomes reported by the studies and a *P*-value less than 0.05 was considered to be statistically significant. We collected the summary OR of NAFLD and low GS. The heterogeneity of results between studies was determined by the *I*^2^ test (22). For *I*^2^, values of 25 to <50% were considered low heterogeneity, 50 to <75% moderate, and 75% highly heterogeneous. If significant heterogeneity was not present (*I*^2^ < 50%), a fixed-effect model was used to pool outcomes, otherwise a random-effect model was applied for the meta-analysis (*I*^2^ > 50%). The publication bias was assessed qualitatively by a visual estimate of the funnel plot and quantitatively by calculation of Begg’s test and Egger’s test (23).

### Subgroup analyses and sensitivity analyses

Subgroup analyses were performed according to the method of GS ascertainment [GS and relative grip strength (RGS)], diagnosis of NAFLD [ultrasonography and hepatic steatosis index (HSI)], region (China and Korea), mean age (<60 and >60 years old), several participants (<5,000 and >5,000). One-study-removed sensitivity analyses were performed to determine the relative impact of each study on the overall risk estimate.

## Results

### Eligible studies and individual characteristics

**FIGURE 1:**
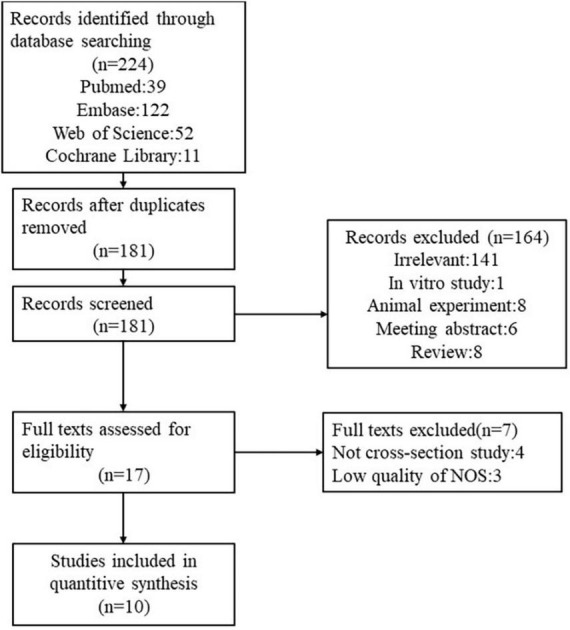
Flow chart of literature search for studies investigating the association between grip strength and NAFLD.

Ten articles were included in this analysis (Figure 1) (9, 24–32). The selected studies involved 76,676 participants. Among the 10 studies, 7 studies (9, 24–29) reported the odds rate (OR) of NAFLD between low GS group and normal group, 4 studies (26, 30–32) reported the OR of low GS between NAFLD group and normal group and 1 studies (26) involved above the two types of OR. The characteristics of the studies included in our meta-analysis are listed in Table 1.

**TABLE 1:** Baseline characteristics of studies included meta-analysis.

Study	Country	Design	Participants	Mean age	OR	Level of quality
Meng et al. (28)	China	Cross-section	20,957	41.2	NAFLD	8
Lee et al. (25)	Korea	Cross-section	538	74.3	NAFLD	7
Lee (26)	Korea	Cross-section	8,001	49.9	NAFLD, LGS	7
Kim et al. (30)	Korea	Cross-section	4,103	60	LGS	7
Gan et al. (24)	China	Cross-section	3,536	53.4	NAFLD	8
Hao et al. (32)	China	Cross-section	1,126	36.5	LGS	5
Park et al. (29)	Korea	Cross-section	3,922	45.9	NAFLD	8
Lee (31)	Korea	Cross-section	1,690	14	LGS	6
Cho et al. (9)	Korea	Cross-section	5,272	57	NAFLD	6
Lee et al. (27)	Korea	Cross-section	27,531	47	NAFLD	7

### Quality of the individual studies

**FIGURE 2:**
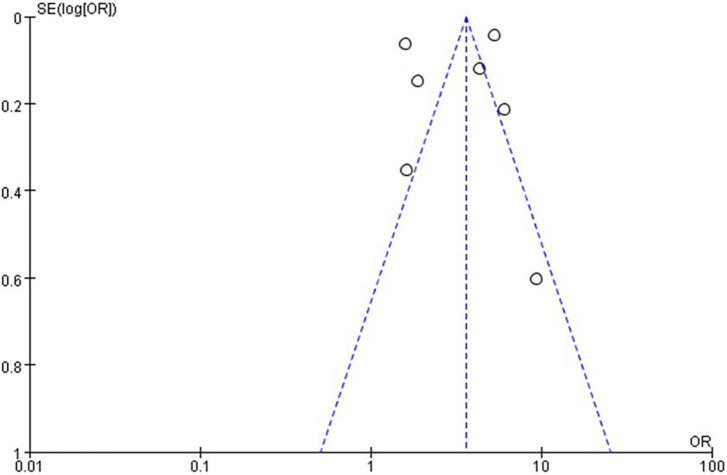
Funnel plot with grip strength and risk of NAFLD.

**FIGURE 3:**
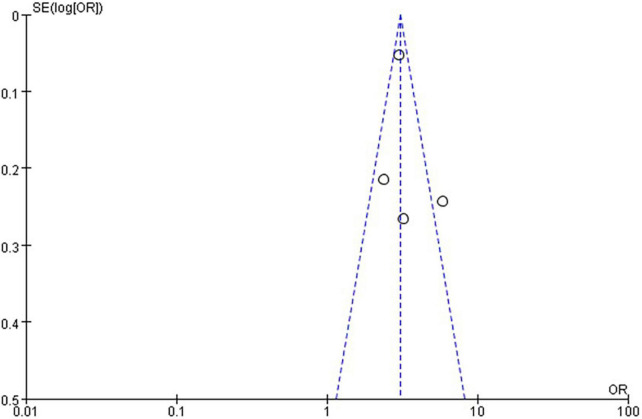
Funnel plot with NAFLD and risk of low grip strength.

The quality level of each study ranged from 5 to 8 stars (Figure 1). The funnel plot (Figures 2, 3) provided a qualitative estimation of publication bias.

### Odds rate of non-alcoholic fatty liver disease between low grip strength group and normal group

**FIGURE 4:**
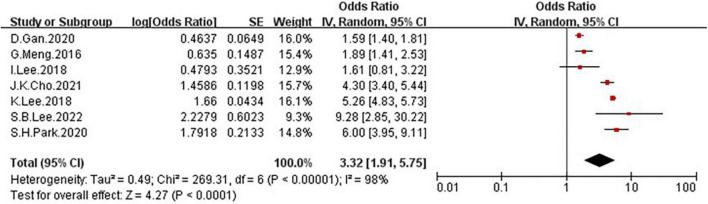
Forest plots showing the relation between low grip strength and risk of NAFLD.

In the seven studies of the OR of NAFLD included in this meta-analysis (Table 1), the sample size varied from 538 to 27,531 participants, and the age varied from 18 to 80 years old. As shown in Figure 4, high heterogeneity was present among the seven studies reporting OR (*I*^2^=98%), so we chose the random-effects model. Meta-analysis of these studies showed that low GS patients had odds of NAFLD that were 3.32 times as high as normal GS (summary OR = 3.32, 95% CI: 1.91–5.75, Figure 4). The result of Funnel plot analysis is showed in Figure 2, and the result of Begg’s test (*P* = 1) and Egger’s test (*P* = 0.785) suggest that there is no significant publication bias.

**FIGURE 5:**
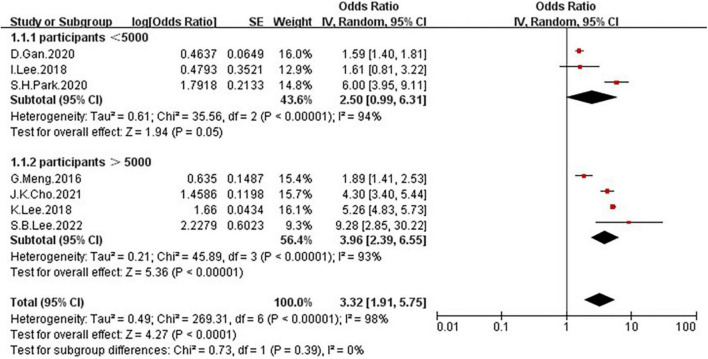
Forest plots depicting the association of low grip strength and the risk of NAFLD were subgrouped by several participants.

**FIGURE 6:**
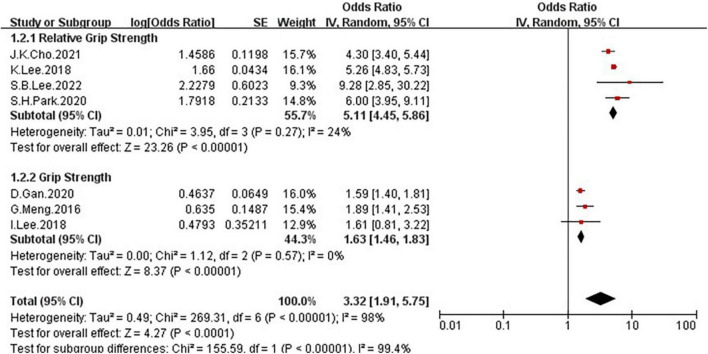
Forest plots depicting the association of low grip strength and the risk of NAFLD were subgrouped by the method of grip strength ascertainment.

**FIGURE 7:**
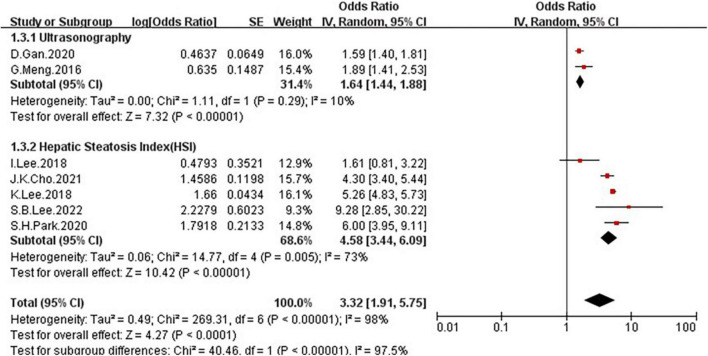
Forest plots depicting the association of low grip strength and the risk of NAFLD were subgrouped by the diagnosis of NAFLD.

**FIGURE 8:**
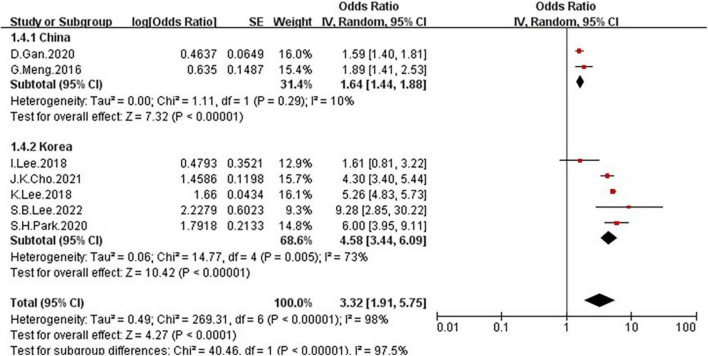
Forest plots depicting the association of low grip strength and the risk of NAFLD were subgrouped by the region.

**FIGURE 9:**
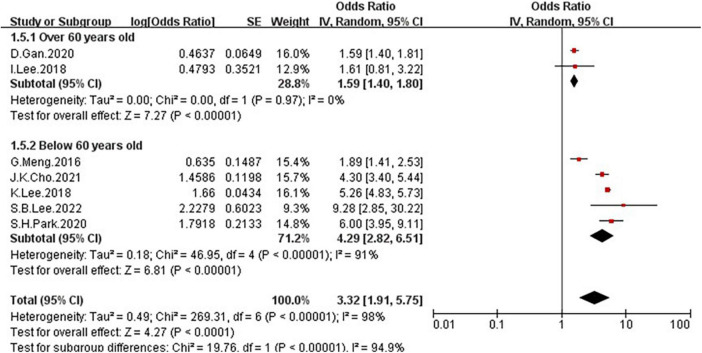
Forest plots depicting the association of low grip strength and the risk of NAFLD were subgrouped by the mean age.

Because of the high heterogeneity, we conducted a series of subgroup analyses to identity the heterogeneity source. The subgroup analysis by several participants revealed no significant difference between numbers (Figure 5), the OR = 2.5, 95% CI: 0.99–6.31 for studies conducted in the number of participants less than 5,000, and OR = 3.96, 95% CI: 2.39–6.55 for studies conducted in several participants more than 5,000. There was a significant association between low GS and risk of NAFLD detected in the studies using RGS (Figure 6, OR = 5.11, 95% CI: 4.45–5.86) compared to the using GS (OR = 1.63, 95% CI: 1.46–1.83). Besides, a significantly greater effect size was observed in the studies using HSI (Figure 7, OR = 4.58, 95% CI: 3.44–6.09) than in the ones applying ultrasonography (OR = 1.64, 95% CI: 1.44–1.88). And the subgroup analysis by region revealed stronger association between GS and risk of NAFLD in the studies in Korea (Figure 8, OR = 4.58, 95% CI: 3.44–6.09) than the studies in China (OR = 1.64, 95% CI: 1.44–1.88). Regarding mean age, compared to the studies with mean age more than 60 years old (Figure 9, OR = 1.59, 95% CI: 1.4–1.8), the studies with mean age of fewer than 60 years old (OR = 4.29, 95% CI: 2.82–6.51) were more strongly associated with the risk of NAFLD. Because of the limited number of original articles, the data are only from China and Korea, therefore, we speculate that the high heterogeneity may be due to the regional distribution of the data and the small number of included articles. And all the subgroup analyses are presented in Table 2.

**TABLE 2:** Subgroup analysis of low grip strength and the risk of NAFLD.

Subgroup title	Number of studies	Summary OR (95% CI)	*P*-for-difference	*P*-for-heterogeneity	*I*^2^ (%)
Overall	7	3.32 (1.91–5.75)	<0.001	<0.001	98
**Region**					
China	2	1.64 (1.44–1.88)	<0.001	0.29	10
Korea	5	4.58 (3.44–6.09)		<0.001	73
**Number of participants**					
<5,000	3	2.5 (0.99–6.31)	0.39	<0.001	94
>5,000	4	3.96 (2.39–6.55)		<0.001	93
**Mean age**					
<60	5	4.29 (2.82–6.51)	<0.001	<0.001	91
>60	2	1.59 (1.4–1.8)		0.97	0
**Method of GS ascertainment**					
Grip strength	3	1.63 (1.46–1.83)	<0.001	<0.001	0
Relative grip strength	4	5.11 (4.45–5.86)		0.27	24
**Diagnosis of NAFLD**					
Ultrasonography	2	1.64 (1.44–1.88)	<0.001	0.29	10
Hepatic steatosis index (HSI)	5	4.58 (3.44–6.09)		<0.001	73

### Odds rate of low grip strength between non-alcoholic fatty liver disease group and normal group

**FIGURE 10:**
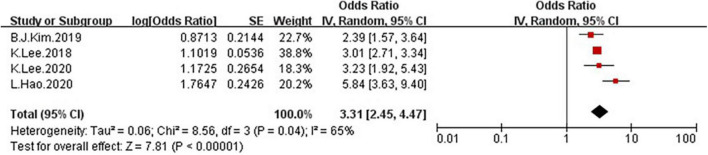
Forest plots showing the relation between NAFLD and risk of low grip strength.

In the four studies of the OR of low GS included in our meta-analysis (Table 1), the sample size varied from 1,126 to 8,001 participants, and the age varied from 10 to 80 years old. As shown in Figure 10, moderate heterogeneity was present among the four studies reporting OR (*I*^2^ = 65%). Meta-analysis of these studies showed that patients with NAFLD had odds of low GS that were 3.31 times as high as normal group (summary OR = 3.31, 95% CI: 2.45–4.47, Figure 10). The result of the funnel plot is presented in Figure 3, and the result of Begg’s test (*P* = 0.734) and Egger’s test (*P* = 0.630) suggest that there is no significant publication bias.

**FIGURE 11:**
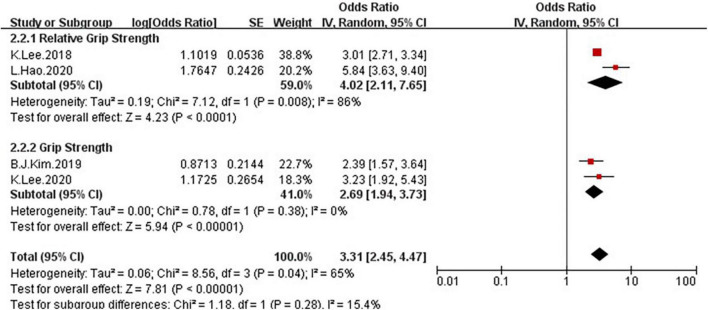
Forest plots depicting the association of NAFLD and the risk of low grip strength were subgrouped by the method of grip strength ascertainment.

Because of the moderate heterogeneity, we conduct a subgroup and meta-regression analyses to identify the heterogeneous source. These four studies, all the shown that NAFLD patients have markedly low GS than the non-NAFLD groups. Because meta-regression was performed to examine possible heterogeneous factors for quantitative variables, we used age as a covariate for meta-regression, but the result (*P* > 0.05) showed that age may not be the cause of high heterogeneity. Additionally, we conducted a subgroup analysis according to the method of GS ascertainment (GS and RGS), there were significant association between NAFLD and low GS was detected in the studies using RGS (Figure 11, OR = 4.02, 95% CI: 2.11–7.65) compared to the studies using GS (OR = 2.69, 95% CI: 1.94–3.73).

### Sensitivity analysis

**FIGURE 12:**
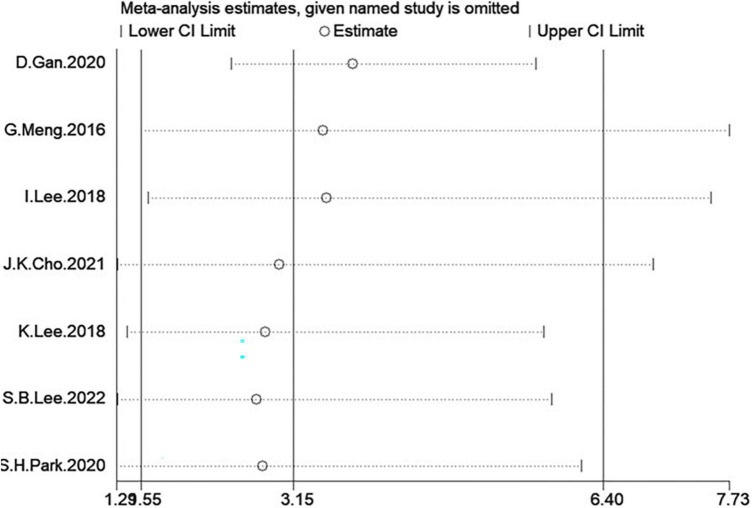
Sensitivity analysis of low grip strength group.

**FIGURE 13:**
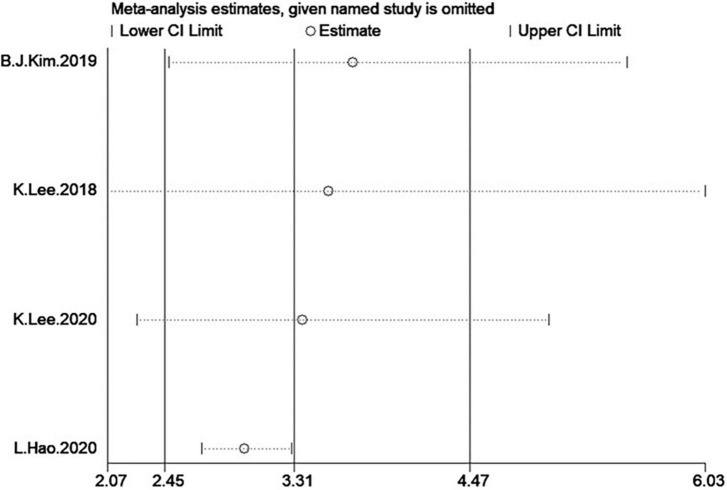
Sensitivity analysis of NAFLD group.

In the two analyses, in one-study-removed sensitivity analyses, we excluded each study and results did not change (Figures 12, 13).

## Discussion

To the best of our knowledge, this is the first systematic review and meta-analysis that summarized available studies regarding the association between GS and NAFLD. In this meta-analysis which included 10 studies with a total of 76,676 participants, we performed two types of meta-analysis, and explored the OR of NAFLD in patients with low GS and the OR of low GS among patients with NAFLD, both results suggest a significant association between the NAFLD and GS: the Figure 4 shown that a significantly increased risk of NAFLD among individuals with low GS with the pooled OR of 3.32 (95% CI: 1.91–5.75). And Figure 10 suggested that NAFLD patients had odds of low GS that were 3.31 times as high as normal people (summary OR = 3.31, 95% CI: 2.45–4.47).

Non-alcoholic fatty liver disease encompasses a wide range of diseases from simple steatosis, non-alcoholic steatohepatitis, fibrosis, and even cirrhosis (33). Skeletal muscle is an insulin-responsive and important endocrine organ, because it secretes myokines that influence metabolic processes in liver and muscle (34). Previously, many reliable studies have found that association between skeletal muscle and NAFLD, Guo et al. (35) reported that skeletal muscle index (SMI) is independently associated with the severity of hepatic steatosis and liver fibrosis of related to NAFLD, and they assessed the association of SMI tertiles with NAFLD and liver fibrosis, individuals with low muscle mass were significantly correlated with NAFLD and liver fibrosis. These findings suggest that NAFLD is affected by skeletal muscle even when people do not have sarcopenia. GS is also an important indicator in the assessment of skeletal muscle and sarcopenia. Previously, several studies have shown a link between sarcopenia and NAFLD, mainly due to a common pathological mechanism, insulin resistance and chronic inflammation have been the most frequently proposed mechanisms, and both are hypothetically plausible (14, 36). Firstly, both the liver and muscle are the target organs for insulin action, and insulin resistance is known as a key factor in the pathophysiology of both NAFLD and sarcopenia (37). With aging, the fat mass in muscle cells increases, which becomes a risk factor for insulin resistance (38). Furthermore, ectopic fat accumulation in the liver is closely associated with systemic insulin resistance (37). Hong (39) reported that an increased insulin resistance index in subjects with sarcopenia compared to those without sarcopenia, and insulin resistance and SMI showed a significant negative correlation, and they also found a significant relationship between insulin resistance and liver attenuation index (LAI), which reflects fat accumulation in the liver.

On the other hand, chronic inflammation is the other hypothesis most often cited. There are several studies focus on the mediators that link the muscle-liver-adipose tissue axis (40). For example, myostatin, a transforming growth factor (TGF)-β superfamily member, is a regulator of skeletal muscle mass, and now some animal studies have shown that myostatin has significant hepatic effects by regulating skeletal muscle metabolism, and blocking myostatin not only increases muscle mass but also protects mice from fatty liver and improves insulin resistance (41). It has been demonstrated that oxidative stress and proinflammatory cytokines of chronic inflammation, such as tumor necrosis factor (TNF)-α and Interleukin (IL)-6 can promote fat and muscle metabolism, leading to loss of skeletal muscle (42), various inflammatory factors released from visceral adipocytes can also promote the development of metabolic syndrome (43), and myonectin and irisin have been suggested to contribute to the development of insulin resistance and fatty liver (44, 45), and Hong (39) also found that high-sensitivity C-reactive protein (hsCRP) concentrations were closely correlated with SMI and LAI, which suggests that inflammation may be an important underlying factor associated with both sarcopenia and NAFLD.

Recently, low vitamin D levels have been suggested to be associated with NAFLD and muscle strength. Vitamin D plays an important role in muscle mass and muscle strength. A systematic review revealed that vitamin D supplementation significantly increased muscle strength (46). A separate study also showed that muscle nuclear vitamin D receptor (VDR) was increased by 30% and augmented muscle fiber size by 10% in elderly females taking vitamin D (47). The involvement of vitamin D in mediating several immune-inflammatory (48) and metabolic processes (49) has been demonstrated previously. Roth et al. (50) reported that vitamin D deficiency exacerbates NAFLD through Toll-like receptors (TLR)-activation in a westernized diet rat model, which causes insulin resistance, higher hepatic resistance gene expression, and up-regulation of hepatic inflammatory and oxidative stress. In humans, hepatic VDR expression is inversely correlated with steatosis severity (51). A recent study (52) have shown that liver VDR expression plays an important role in regulating intra-hepatic lipid accumulation.

In addition, gut microbiota is also an important component in the pathological mechanism. The gut microbiota composition is generally shaped in early childhood (53), and by the age of three years old (54), the gut microbiota reaches its mature composition, which is maintained relatively stable over the lifespan, and after age of 65, gut microbiota resilience is generally reduced. In current studies, there is evidence supporting the concept that the gut microbiota composition is moderated by exercise (55), including the animal models (56) and human studies (57). Currently, the most studied putative mediators of the effect of gut microbiota on skeletal muscle function are short-chain fatty acids (SCFA) (58), and the SCFA produced by gut microbiota can enter systemic circulation and be absorbed by skeletal muscle cells, where they act as ligands for free fatty acids receptors 2 and 3 (59), and these receptors have a key role in moderating glucose uptake and metabolism, and in promoting insulin sensitivity (60). In addition, gut microbiota also plays a role in NAFLD. As we all know, increased dietary fat intake, is associated with the development of NAFLD (61), and the high fat diet can alter the gut microbiota, and favoring gut bacteria associated with the development of NAFLD (62).

This study also has several limitations. Firstly, one analysis has moderate heterogeneity, and another analysis has high heterogeneity, which maybe because of the small number of included studies, the restricted regional distribution of the studies, and age differences in each study. Secondly, in the subgroup analyses, some subgroups only have 2 or 3 studies, which may affect the results. Besides, subgroup analyses are observational by nature and may be subject to confounding by study-level characteristics. Finally, the definition of NAFLD is different in included studies, some studies use ultrasound to examine the NAFLD, and some studies use HSI to diagnose NAFLD, which may affect the study findings.

Therefore, GS, as an important parameter of sarcopenia and muscle strength, is associated with NAFLD, not only in pathological mechanisms, such as insulin resistance, chronic inflammation, gut microbiota, and regulation of vitamin D, but also in terms of clinical data that people with low GS have a higher risk of NAFLD, and patient with NAFLD have lower GS than normal people.

## Conclusion

In conclusion, there is an association between NAFLD and GS. Compare with the normal group, people with low GS are more likely to develop NAFLD, in addition, GS levels in NAFLD patients are also generally lower than the normal population.

## Data availability statement

The original contributions presented in this study are included in the article/Supplementary material, further inquiries can be directed to the corresponding author.

## Author contributions

LH: study idea, concept and design, data extraction and interpretation of data, drafting of the manuscript, and review of the final manuscript. SF: data extraction and analysis of data, drafting of the manuscript, and review of the final manuscript. JL: drafting of the manuscript, data analysis, and review of the final manuscript. DL: drafting of the manuscript and review of the final manuscript. YT: study idea, concept and design, drafting of the manuscript, and review of the final manuscript. All authors contributed to the article and approved the submitted version.
