# Supplementary material for: Association between grip strength and non-alcoholic fatty liver disease: A systematic review and meta-analysis
Source: Front Med (Lausanne). 2022 Aug 26;9:988566. doi: 10.3389/fmed.2022.988566 (PMC9458919; doi:10.3389/fmed.2022.988566)
Supplement: Supplementary file 1 [file Table_1.pdf]

Supplemental table 1. The search strategy for each database

Pubmed	#1 ("Non-alcoholic Fatty Liver Disease"[Mesh]) OR (((((((((((Non alcoholic Fatty Liver Disease) OR (Nonalcoholic Fatty Liver Disease)) OR (Fatty Liver, Nonalcoholic)) OR (Fatty Livers, Nonalcoholic)) OR (Liver, Nonalcoholic Fatty)) OR (Livers, Nonalcoholic Fatty)) OR (Nonalcoholic Fatty Liver)) OR (Nonalcoholic Fatty Livers)) OR (Nonalcoholic Steatohepatitis)) OR (Nonalcoholic Steatohepatitides)) OR (Steatohepatitides, Nonalcoholic)) OR (Steatohepatitis, Nonalcoholic))
	#2 ("Hand Strength"[Mesh]) OR (((((((((Strength, Hand) OR (Grip Strength)) OR (Strength, Grip)) OR (Hand Grip Strength)) OR (Grip Strength, Hand)) OR (Strength, Hand Grip)) OR (Grip)) OR (Grips)) OR (Grasp)) OR (Grasps))
	#3 #1 AND #2
Web of Science	#1 TS=(Hand strength OR Strength, Hand OR Grip Strength OR Strength, Grip OR Hand Grip Strength OR Grip Strength, Hand OR Strength, Hand Grip OR Grip OR Grips OR Grasp OR Grasps)
	#2 TS=(Non-alcoholic Fatty Liver Disease OR Non alcoholic Fatty Liver Disease OR NAFLD OR Nonalcoholic Fatty Liver Disease OR Fatty Liver, Nonalcoholic OR Fatty Livers, Nonalcoholic OR Liver, Nonalcoholic Fatty OR Livers, Nonalcoholic Fatty OR Nonalcoholic Fatty Liver OR Nonalcoholic Fatty Livers OR Nonalcoholic Steatohepatitis OR Nonalcoholic Steatohepatitides OR Steatohepatitides, Nonalcoholic OR Steatohepatitis, Nonalcoholic)
	#3 #1 AND #2
Cochrane Library	#1 MeSH descriptor: [Hand strength]

	explode all trees
	#2 (strength, Hand OR Grip Strength OR Strength, Grip OR Hand Grip Strength, Hand OR Strength, Hand Grip OR Grip OR Grasp OR Grasps)
	#3 MeSH descriptor: [Non-alcoholic Fatty Liver Disease] explode all trees
	#4 (Non alcoholic Fatty Liver Disease OR NAFLD OR Nonalcoholic Fatty Liver Disease OR Fatty Liver, Nonalcoholic OR Fatty Livers, Nonalcoholic OR Liver, Nonalcoholic Fatty OR Livers, Nonalcoholic Fatty OR Nonalcoholic Fatty Liver OR Nonalcoholic Fatty Livers OR Nonalcoholic Steatohepatitis OR Nonalcoholic Steatohepatitides OR Steatohepatitides, Nonalcoholic OR Steatohepatitis, Nonalcoholic)
	#5 #1 OR #2
	#6 #3 OR #4
	#7 #5 AND #6
Embase	#1 ("Non-alcoholic Fatty Liver Disease"[Mesh]) OR ((((((((((((Non alcoholic Fatty Liver Disease) OR (Nonalcoholic Fatty Liver Disease)) OR (Fatty Liver, Nonalcoholic)) OR (Fatty Livers, Nonalcoholic)) OR (Liver, Nonalcoholic Fatty)) OR (Livers, Nonalcoholic Fatty)) OR (Nonalcoholic Fatty Liver)) OR (Nonalcoholic Fatty Livers)) OR (Nonalcoholic Steatohepatitis)) OR (Nonalcoholic Steatohepatitides)) OR (Steatohepatitides, Nonalcoholic)) OR (Steatohepatitis, Nonalcoholic))
	#2 ("Hand Strength"[Mesh]) OR ((((((((((Strength, Hand) OR (Grip Strength)) OR (Strength, Grip)) OR (Hand Grip Strength)) OR (Grip Strength, Hand)) OR (Strength, Hand Grip)) OR (Grip)) OR (Grips)) OR (Grasp)) OR (Grasps))
	#3 #1 AND #2
